# The Symptom Burden and Quality of Life in Cancer Patients in the Gaza Strip, Palestine: A Cross-Sectional Study

**DOI:** 10.1371/journal.pone.0262512

**Published:** 2022-01-13

**Authors:** Yousuf ElMokhallalati, Enas Alaloul, Mohammed Shatat, Tasneem Shneewra, Saad El Massri, Omar Shaer, Samuel Relton, Hammoda Abu-Odah, Matthew J. Allsop

**Affiliations:** 1 Leeds Institute of Health Sciences, School of Medicine, University of Leeds, Leeds, United Kingdom; 2 Ministry of Health, Gaza, occupied Palestinian territory; 3 Faculty of Medicine, Islamic University, Gaza, occupied Palestinian territory; 4 School of Nursing, The Hong Kong Polytechnic University, Hung Hom, Hong Kong; University of Auckland, NEW ZEALAND

## Abstract

**Objectives:**

Cancer is the second leading cause of death in the Gaza Strip, Palestine, but there is an absence of evidence systematically assessing symptom burden and quality of life (QoL) using validated tools. Our objective was to assess associations between socio-demographic and disease-related characteristics, symptom burden and QoL in a sample of cancer patients accessing outpatient services in the Gaza Strip.

**Design:**

A cross-sectional, descriptive survey using interviews and medical record review involving patients with cancer accessing oncology outpatient services at Al Rantisi Hospital and European Gaza Hospital (EGH) in the Gaza Strip was employed. Socio-demographic and disease-related data, the Lebanese version of the Memorial Symptom Assessment Scale (MSAS-Leb), and the Arabic version of the European Organization for Research and Treatment of Cancer Quality of Life Questionnaire-C30 (EORTC QLQ-C30) were collected. Multiple linear regression was used to judge the relative influence of determinants of QoL.

**Results:**

Of 414 cancer patients approached, 385 patients consented to participation. The majority were women (64.7%) with a mean age of 52 years (SD = 16.7). Common cancer diagnoses were breast (32.2%), haematological (17.9%) and colorectal (9.1%). The median number of symptoms was 10 (IQR 1.5–18.5). Mean overall QoL was 70.5 (SD 19.9) with common physical and psychological symptoms identified. A higher burden of symptoms was associated with marital status, education and income. Limited access to both opioids and psychological support were reported.

**Conclusions:**

A high symptom burden was identified in outpatients with cancer. Increasing provision and access to supportive care for physical and psychological symptoms should be prioritised alongside exploring routine assessment of symptom burden and QoL.

## Introduction

Palestine, along with other LMICs, is experiencing a growth in the burden of non-communicable diseases [1, 2]. Cancer is the second leading cause of morbidity and mortality in Palestine, exceeded only by cardiovascular disease [3]. The most common type of cancers among adult patients in the Gaza Strip are breast, colorectal, and lung cancers [3]. The health system in Palestine is largely fragmented and under-resourced and experiencing issues including a lack of effective governance, evidence-based policies, and financing [4]. Furthermore, due to ongoing conflict surrounding Israeli occupation, there are frequent reports of damage to health facilities, alongside health staff and civilians being injured and killed [5]. Since 2018, direct Israeli attacks have caused 48,246 injuries in Palestine and 452 fatalities (the majority in the Gaza-Strip) [5]. In the context of the Gaza Strip, the complex and ongoing socio-political and economic crises faced by the Ministry of Health heavily affect the delivery of care for people with cancer. Whilst affecting all areas of healthcare delivery, the chronic shortage of many essential medicines arising through political instability and a lack of funding, leads to 30–40% of essential chemotherapy drugs not being in stock at any one point in time. This can lead to delays in receiving treatment and missed doses, leading to a worse prognosis and increased mortality for patients [6].

Across all cancer types, the disease can have a negative influence on a patient’s physical, social, mental, and emotional well-being [7, 8]. Furthermore, the diagnosis of cancer can present varied and multi-dimensional issues that may be problematic for a patient, including affecting existing relationships with partners, causing trauma to children of adults with cancer, and increasing the risk of impairments to psychological and physical health for patients, their caregivers and their families [9, 10]. Furthermore, cancer treatment can give rise to symptoms and side effects that may persist beyond treatment, increasing physical and psychological distress, anxiety and depression, which may decrease quality of life [11, 12]. Despite fragmented care and the increasing burden of cancer in the Gaza Strip, there has been no reporting of the impact of living with and undergoing treatment for cancer that utilise validated measures of, for example, patient outcomes and experiences. Increasingly, patient-reported outcome measures are being incorporated into cancer care, seeking to put the patient and their needs at the centre of decision making about their care [13]. Measures such as those for quality of life can help to capture an individual’s perception of their position in life in the context of the culture and value systems in which they live and concerning their goals, expectations, standards and concerns [14].

In the Gaza Strip, the limited literature available regarding the provision of cancer care suggests that patients with cancer report a reduced quality of life [15]. However, to date, there has been no comprehensive assessment of which factors may influence the quality of life for patients with cancer. In particular, the experience of people with cancer living in the community is unclear, including, for example, the extent of symptom burden. This study seeks to address this gap and aimed to determine symptom burden and factors associated with quality of life in a sample of cancer patients accessing outpatient services in the Gaza Strip. The collection of data on cancer patients that makes use of validated measures can provide insights into the experience of patients and direct priorities for the future development of oncology and palliative care services in Gaza.

## Methods

### Study design

A cross-sectional, descriptive survey design was conducted, using a structured questionnaire to collect data from patients with cancer attending outpatient clinics in two oncology hospitals in the Gaza Strip. A team of researchers from the Ministry of Health and Islamic University, both based in Gaza, conducted the data collection between July and August 2019.

### Study population

In this study, the target population was patients with cancer who accessed oncology outpatient services which provide age-appropriate treatment patients at Al Rantisi Hospital and European Gaza Hospital (EGH) in Gaza Strip. These are the main government-funded sites for the provision of cancer care in the Gaza Strip.

### Eligibility criteria

**Inclusion criteria**.

- Patients with cancer aged 13 years and over who attended oncology outpatient services at Al Rantisi Hospital and European Gaza Hospital. In Gaza, those aged 13 years and over access treatment through adults oncology services at the two oncology hospitals.
- Patients who were able to respond to the questionnaire.

**Exclusion criteria**.

- Children aged below 13 years who attended paediatric oncology outpatient services at Al Rantisi Hospital and European Gaza Hospital.

### Study setting and period

This study has been conducted at the main two oncology departments (Al Rantisi Hospital and EGH) in Gaza Strip during July and August 2019.

### Sample and sample size calculation

Members of the research team (MS, TS, SM, OS) attended each outpatient oncology clinic at Al Rantisi Hospital and EGH in Gaza Strip during July and August 2019 using convenience sampling until achieving the study target sample. Outpatient oncology clinics led by the two hospital sites include disparate patient cohorts, with multiple cancer types across disease stages.

To inform sample size calculations, for the EORTC QLQ-C30, as well as subscale specific scores, you can calculate an overall global health status/QoL score. From the EORTC QLQ-C30 manual, we assumed this is normally distributed with a mean of 61.3 and SD = +/- 24.2. Assuming an SD of 25, to estimate the mean QoL score with a 95% CI (t-based) with +/- 5 precision requires 100 participants (e.g. if you have 100 participants and their mean total QoL score is 61.3 then the 95% CI will be 56.3, 66.3). For the MSAS-Leb, similarly as well as the subscale specific scores, you can calculate an overall total MSAS score. A previous study [16] provides data for the tool in cancer patients with a mean of 2.36 and SD of +/- 0.59. Therefore, assuming an SD of 1 (conservative assumption), to estimate the total MSAS with a 95% CI (t-based) with +/- 0.2 precision requires 100 participants (selected the precision to give the same sample size as above), e.g. if you have 100 participants and the mean total MSAS score is 2.36 then the 95% CI will be 2.16, 2.56. Both tools also include various subscale outcomes on the proportion/percentage scale. Using the Statulator tool (http://statulator.com/SampleSize/ss1P.html), to ensure we had an adequate sample size to robustly estimate these, we determined that to estimate any proportion (or %) with 5% absolute precision (i.e. +/- 5 percentage points) requires 385 people.

### Assessments tools

A structured questionnaire was used in this study which consisted of the following parts: participants’ socio-demographic and disease-related characteristics, Memorial Symptom Assessment Scale and the European Organization for Research and Treatment of Cancer Quality of Life Questionnaire. See S1 Appendix for the questionnaire and its content.

#### Socio-demographic and disease-related characteristics

Socio-demographic characteristics collected using a structured questionnaire included participants age, sex, marital status, education level, monthly income and residency. In addition, we collected information on the participants’ condition including cancer diagnosis, length of illness, disease stage, referral to the cancer centre and comorbidities (hypertension, diabetes, heart disease, dyslipidaemia) and current/prior treatment (treatment modalities and current pain medications). Items relating to sociodemographic and clinical information (e.g. time since diagnosis, treatments accessed) were developed specifically for this study. To ensure their meaning and ease of readability, the items were piloted with members of the research team not involved in the development of the tool. This enabled the opportunity to determine whether items were clear and could be interpreted. Following testing, the research team met to discuss the questionnaire and its content, to ensure consistency in its use during data collection.

#### Symptom burden

The Lebanese version of the Memorial Symptom Assessment Scale (MSAS-Leb) [17] was utilised to measure 30 psychological and physical symptoms in terms of prevalence, severity, and distress in the last week. A symptom score is derived from the average of three symptom dimensions; prevalence, severity, and distress within the last week, where a higher average score equates to a greater symptom burden [17, 18]. The total score of MSAS-Leb is the average score of all 30 symptoms [17, 19]. The MSAS has been widely used and validated in different cancer populations. MSAS-Leb, which was adopted from MSAS, was validated in the outpatient oncology setting [17]. The original version of MSAS contains 32 symptoms. Two items, feeling irritable and feeling drowsy, were removed in the MSAS-Leb because of their respective similarity in Arabic to another two items in the scale, namely feeling nervous and dizziness. Thus, the final version of the MSAS-Leb contains 30 symptoms.

#### Health-related quality of life

The Arabic version of the European Organization for Research and Treatment of Cancer Quality of Life Questionnaire-C30 (EORTC QLQ-C30) [20] was used to measure the quality of life of cancer patients. The questionnaire measures five function scales, three symptom subscales and six single-item scales and one global health status (GHS). Subscale scores ranged from 0 to 100, where higher scores for functional scales and global health status represent better QoL and lower scores for symptom scales represent less burden and less symptom related issues [21]. The Arabic version of EORTC QLQ-C30 has been validated in a wide range of inpatient and outpatient clinical settings [21–23].

### Procedure

All participants were provided with an information sheet about the study aims at arrival to an outpatient clinic by a member of the research team responsible. Clinicians who conducted the routine consultations, who were not members of the research team, discussed the study with potential participants and gauged interest and willingness to participate. Participants were given the opportunity to review study details ahead of their planned consultation. All participants provided consent prior to participation. For participants under the age of 18, the study was first discussed with a parent or guardian. If a parent or guardian consented to the research team approaching the eligible participant under 18 years of age, a member of the study team discussed the study, provided an information sheet and obtained additional consent from the participant. A parent or guardian was present during all study activities for participants under 18 years of age. All participants who chose to take part in this study were accompanied by a member of the research team (MS, TS, SM, OS) team to a separate, private room close to the outpatient clinic to complete a standardised questionnaire. The standardised questionnaire included validated tools for measuring symptom burden and quality of life and captured sociodemographic and clinical information. During data collection, to support participant recall of dates relating to the length of illness and presentation to cancer centres, researchers orientated participants and explored proximity to significant dates (e.g. family/respondent’s birthdays, or religious holidays) to increase accuracy. Furthermore, the research team member subsequently reviewed the medical records of participants to verify the information and complete any data that participants were not able to recall (e.g. date of initial referral to the outpatient clinic). Written informed consent was obtained from all participants.

### Statistical analysis

Descriptive analysis was used to summarise socio-demographic data, disease-related data, and summary scores for the MSAS-Leb and EORTC QLQ-C30. Mann–Whitney U-test was used for between-subjects comparisons. To compare our study results with previous studies [17, 19] MSAS-Leb scores are presented as mean (SD) as aligned with previous analyses, although the variables were often skewed.

To assess the association between QOL and socio-demographic and disease-related characteristics, automatic linear regression was used with forward stepwise model selection and variables entered and removed at the 0.1 significance level. Regression analysis results are presented as unstandardized beta coefficients with 95% confidence intervals (CIs), corresponding P-value and predictor importance. Predictor importance indicates the relative importance of each predictor on the constructed model (measured from 0 to 1).

The total score of EORTC QLQ-C30 was the dependent variable. Covariates included gender, age in years, educational level (less than secondary school, secondary school, university degree or more), hospital site (Al Rantisi Hospital, EGH), marital status (married, unmarried), comorbid conditions (yes, no), disease stage at referral (early stages, advanced stages), monthly income (NIS) (< 1000, 1000–2000, >2000), the total MSAS-Leb score, receiving chemotherapy (yes, no) and receiving radiation (yes, no). Complete case analysis was conducted as there was very little missing data. For all analyses, p <0.05 was considered statistically significant. SPSS software (version 26) was used for data analysis.

### Ethical review

Ethical approval was obtained from the Palestinian Ministry of Health (ref: 329501) and the School of Medicine Ethics Committee at the University of Leeds, UK (ref: MREC 18–092).

## Results

A total of 414 participants were approached, assessed and confirmed as eligible to participate and invited to participate in the study. A total of 385 participants took part (92.9% response rate) in both Al Rantisi Hospital (n = 255; 66.2%) and European Gaza Hospital (n = 130;33.8%) (see Table 1 for an overview of participant characteristics). The majority of the participants (64.7%) were female. Mean age of participants was 51.6 (SD = 16.8). Breast (n = 124; 32.2%), hematological (n = 69;17.8%) and colorectal (n = 35; 9.1%) were the most common cancer types. Stage of disease at the point of referral to outpatient services included both early (n = 268;69.8%) and late (n = 107;27.9%). Treatment modes received in the last 3 months included chemotherapy (n = 248;64.4%), surgery (n = 101;26.2%) and radiation (n = 76;19.7%), with 297 (77.1%) participants reporting access to pain medication. Data were missing for the following variables: educational level (n = 1; 0.3%), age (n = 1; 0.3%), date of primary cancer diagnosis (n = 3;0.8%), disease stage (n = 3;0.8%), MSAS scores (n = 14;3.6%), and EORTC QLQ-C30 scores (n = 3;0.8%).

**Table 1:** Overview of participant characteristics.

	Participant characteristics	Total = n (%)
	**Study population**	385 (100)
**Socio-demographic characteristics**		
	Female	249 (64.7)
	Male	136 (36.3)
	**Age**	
	<18	16 (4.2)
	18–44	69 (18.0)
	45–64	157 (40.8)
	65+	142 (36.8)
	**Educational level**	
	Less than secondary school	157 (40.9)
	Secondary school	124 (32.3)
	University degree or more	103 (26.8)
	**Monthly income (NIS)**	
	< 1000	224 (58.2)
	1000–2000	95 (24.7)
	2000–3000	25 (6.5)
	>3000	11 (2.8)
	Not applicable/ I prefer not to say	30 (7.8)
	**Residency**	
	Outside camp	296 (76.9)
	Inside camp	89 (23.1)
**Disease characteristics**	**Primary cancer diagnosis leading to cancer centre referral**	
	Breast	124 (32.2)
	Haematological	69 (17.9)
	Colorectal	35 (9.1)
	Head and neck	32 (8.3)
	Lung	23 (6.0)
	Prostate	18 (4.7)
	Upper gastrointestinal (upper GI)	16 (4.2)
	Brain	14 (3.6)
	Other	13 (3.4)
	Soft connective tissues	12 (3.1)
	Liver	11 (2.9)
	Urological	7 (1.8)
	Female genital and reproductive organs	6 (1.6)
	Skin	4 (1.0)
	Other male genital and reproductive organs (excluding prostate)	1 (0.2)
	**Date of primary cancer diagnosis prior to participation**	
	< 6 months	110 (28.8)
	6–12 months	73 (19.1)
	12–24 months	30 (7.9)
	> 24 months	169 (44.2)
	**Disease stage at referral**	
	Early stages	268 (69.6)
	Advanced stages	107 (27.8)
	Unavailable / I don’t know	7 (1.8)
	**Comorbid conditions**	
	No	228 (59.5)
	1	89 (23.1)
	>1	67 (17.4)
**Treatment**	**Treatments modalities received in last 3 months**	
	Chemotherapy	248 (64.4)
	Hormonal	48 (12.5)
	Radiation	76 (19.7)
	Surgical	101 (26.2)
	Psychological	4 (1)
	Other treatment	11 (2.9)
	**Pain medication accessed**	
	Yes	232 (60.3)
	No	153 (39.8)
	**Type of pain medication accessed**	
	Non-opioid drugs	169 (43.9)
	Weak opioid drugs	61 (15.8)
	Strong opioid drugs	64 (16.6)

### Symptom burden

Findings showed that the most commonly reported physical symptoms included ‘Numbness/tingling in hands/feet’ (56%), lack of energy (55%), and pain (52%); the most frequently reported psychological symptoms were feeling nervous (57%), feeling sad (54%), and worrying (53%). Over half of all participants reported each of these physical and psychological symptoms. Specific symptom prevalence and associated distress are presented in Table 2.

**Table 2:** Mean scores of the Memorial Symptom Assessment Scale (MSAS) indicating overall experience (taken from an average of frequency, severity and distress).

		Male		Female		Overall	
Symptom	*Prevalence (%)*	*Mean*	*SD*	*Mean*	*SD*	*Mean*	*SD*
Feeling nervous	57	2.82	0.76	2.88	0.78	2.86	0.77
Numbness/tingling in hands/feet	56	2.84	0.72	2.88	0.76	2.87	0.75
Lack of energy	55	2.96	0.72	2.96	0.82	2.96	0.78
Feeling sad	54	2.86	0.73	2.93	0.75	2.91	0.74
Worrying	53	2.94	0.71	2.96	0.68	2.95	0.69
Pain	52	2.83	0.76	2.92	0.77	2.89	0.76
Dizziness	48	2.68	0.91	2.68	0.81	2.68	0.85
Difficulty sleeping	42	2.62	0.74	2.78	0.71	2.72	0.72
Dry mouth	42	2.80	0.70	2.80	0.72	2.80	0.71
‘‘I don’t look like myself”	40	3.05	0.80	2.99	0.77	3.02	0.78
Weight loss	36	2.82	0.80	2.76	0.71	2.79	0.75
Lack of appetite	35	2.72	0.77	2.77	0.76	2.75	0.76
Difficulty concentrating	34	2.58	0.75	2.68	0.86	2.65	0.82
Shortness of breath	33	2.75	0.79	2.68	0.79	2.71	0.79
Nausea	31	2.48	0.82	2.81	0.71	2.69	0.76
Cough	27	2.72	0.73	2.79	0.80	2.76	0.77
Swelling of arms/legs	27	2.61	0.67	2.62	0.73	2.61	0.71
Problems with urination	26	2.86	0.72	2.91	0.65	2.89	0.68
Constipation	25	2.85	0.66	2.79	0.71	2.82	0.68
Vomiting	22	2.45	1.01	2.64	0.86	2.58	0.91
Hair loss	22	2.39	0.67	2.97	0.70	2.81	0.73
Sweats	21	2.75	0.54	2.91	0.77	2.84	0.67
Mouth sores	20	2.83	0.67	2.82	0.80	2.82	0.74
Itching	19	2.70	0.72	2.81	0.72	2.76	0.72
Change in skin	19	2.61	0.61	2.63	0.51	2.62	0.55
Difficulty swallowing	17	2.87	0.71	2.91	0.58	2.89	0.63
Change in the way food tastes	15	3.07	0.82	2.98	0.83	3.01	0.82
Diarrhoea	14	2.41	0.72	2.69	0.69	2.57	0.71
Feeling bloated	8	2.99	0.73	2.82	0.85	2.87	0.81
Problems with sexual interest	3	3.18	0.95	2.46	0.96	2.70	0.97

Across all symptoms, when present, scores suggested moderate to high levels of distress (Table 3). The high levels of distress and frequency of symptoms were reflected across the MSAS-Leb sub-scales. The mean MSAS-Leb global distress measure indicated overall symptom distress was 2.86 (SD = 0.57). Across the sub-scales, the average of the distress associated with physical symptoms was 2.71 (SD = 0.59), and the average frequency associated with psychological symptoms was 2.75 (SD = 0.65). The average of the symptom scores across all 32 symptoms in the MSAS-Leb was 2.72 (SD = 0.49).

**Table 3:** Socio-demographic characteristics and related symptom distress reported by participants.

	Global distress Index Mean Rank	Physical symptom Index Mean Rank	Psychological Index Mean Rank	Total MSAS (mean rank)
**Total (SD)**	2.8634 (0.57)	2.7141 (0.59)	2.7534 (0.65)	2.7201 (0.49)
**Sex**				
Male	2.887	2.7074	2.7153	2.7089
Female	2.8503	2.718	2.7735	2.7263
**Age**				
<18	2.7587	2.4387	2.7568**	2.6749
18–44	2.9354	2.686	2.9151**	2.7246
45–64	2.8408	2.7136	2.7458**	2.7129
65+	2.8445	2.7974	2.6007**	2.7442
**Educational level**				
Less than secondary school	2.9261	2.7784	2.8198	2.7955**
Secondary school	2.8203	2.6733	2.7482	2.6645**
University degree or more	2.8172	2.6477	2.6632	2.67**
**Hospital site**				
Al Rantisi Hospital	2.8353	2.6189**	2.7452	2.6748**
EGH	2.9202	2.8981**	2.77	2.8084**
**Governorate**				
North Gaza	3.0004	2.7492***	2.8237	2.781**
Gaza	2.7941	2.6338***	2.7166	2.6682**
Middle	2.7489	2.5128***	2.7077	2.5811**
Khanyounis	2.9548	2.8593***	2.8056	2.8173**
Rafah	2.9084	2.9201***	2.7583	2.8163**
**Marital status**				
Married	2.8466**	2.7171**	2.7267	2.7067
Single	2.8639**	2.5966**	2.8375	2.7162
Divorced / separated / Widowed / Widower	3.2236**	3.0575**	3.0313	3.0021
**Monthly income (NIS)**				
< 1000	2.9392**	2.7904**	2.826***	2.8036***
1000–2000	2.7895**	2.6325**	2.6572***	2.6074***
2000–3000	2.6493**	2.4713**	2.5103***	2.5635***
>3000	2.502**	2.4619**	1.8937***	2.3862***

*** p <0.001

** p <0.01; * p <0.05.

### Health-related quality of life

The overall quality of life score reported across participants differed very little between men and women (see Table 4). The overall quality of life of participants was reported as 70.50 (SD = 19.84). Functional scales indicated variation in quality of life, with physical functioning and role functioning most adversely affected. The burden from symptoms varied, with the greatest burden reported from fatigue (50.70; SD = 29.58), pain (32.51; SD = 31.48) and insomnia (27.36; SD = 32.92).

**Table 4:** The mean scores of Cancer Quality of Life Questionnaire-Core 30 (EORTC QLQ-C30).

	Male		Female		Overall	
	Mean	SD	Mean	SD	Mean	SD
Overall QLQ-30 Score	69.8498	19.53382	70.8475	20.03245	70.4951	19.838
**Global health status**						
Global health status	56.3725	25.03568	60.3079	25.17312	58.9177	25.16262
**Functional Scales**						
Physical functioning	55.8824	28.59749	60.3748	30.72952	58.7879	30.0333
Role functioning	55.8824	33.24243	56.4926	35.31116	56.2771	34.55225
Emotional functioning	64.2157	27.10515	64.257	28.3447	64.2424	27.8777
Cognitive functioning	78.5539	25.9715	76.0375	26.20922	76.9264	26.11942
Social functioning	59.1912	35.1844	66.0643	34.07565	63.6364	34.58242
**Symptom Scales**						
Fatigue	53.3497	27.80887	49.2637	30.45484	50.7071	29.57546
Nausea and vomiting	15.8088	24.53853	16.5997	25.0223	16.3203	24.82336
Pain	32.3529	31.60746	32.5971	31.47569	32.5108	31.48135
Dyspnoea	20.8333	30.61442	20.7497	30.56473	20.7792	30.54243
Insomnia	27.6961	32.84961	27.1754	33.02967	27.3593	32.9243
Appetite loss	24.5098	32.51724	22.3561	31.32355	23.1169	31.72481
Constipation	22.549**	31.65721	13.5207**	26.60882	16.71	28.77964
Diarrhoea	11.0294	24.36186	8.166	21.79822	9.1775	22.74656
**Financial difficulties**						
Financial difficulties	55.1471*	38.7837	46.0509*	39.64194	49.2641	39.53075

All scale scores are linearly converted to range from 0 to 100; for the symptom scales higher scores indicate higher symptom burden.

*** p <0.001

** p <0.01

* p <0.05.

Table 5 presents the results of a multivariate linear regression model which was performed to identify factors associated with QOL. Higher scores of the MSAS- Leb (more symptom burden) and lower educational level was negatively associated with QOL. Participants who did not receive radiotherapy and unmarried participants had better QOL scores. Early stage of cancer was also associated with better QOL scores (*R*2 = 21%).

**Table 5:** Multiple linear regression of factors associated with QOL.

Covariates^a^	B(SE)	95% CI	Importance
**The mean scores of the MSAS- Leb**	-15.6 (1.9)***	-19.5 to -11.9	0.75
**Educational level**			0.73
Less than secondary school versus university degree or more	-5.2 (2.3)**	-9.8 to -0.7	0.73
Secondary school versus university degree or more	-5.5 (2.4)**	-10.3 to -0.7	0.73
**Marital status (unmarried v married)**	5.2 (2.4)*	0.5 to 9.9	0.05
**Disease stage at referral (**early stage v advanced stage)	5.9 (2.1)***	1.8 to 9.9	0.09
**Radiation** (no v yes)	3.8 (2.3)	-0.8 to 8.3	0.03

B unstandardized beta coefficient, SE standard error CI confidence interval

^a^Adjusted *R*^2^ = 21%

*** p <0.001

** p <0.01

* p <0.05.

## Discussion

### Summary of main findings

This is the first study to comprehensively assess the symptom burden and quality of life of patients with cancer in the Gaza Strip. The findings highlighted many unresolved problematic symptoms for participants living with cancer that affect their quality of life. A high level of symptom burden was observed among participants, with fatigue the commonest, alongside a high prevalence of symptoms aligned with emotional and social scales. This study also highlights that, despite reporting access to pain medication, over half of the participants reported high levels of pain. Furthermore, physical symptom burden and psychological symptom burden were independently associated with lower QoL. Through the systematic use of validated measures to determine symptom burden and quality of life, our findings contribute to a limited evidence base to inform a necessary response to support people with cancer in the Gaza Strip.

### Comparison with existing literature

Participants in this study reported symptom burden at higher levels than typically documented in patients with cancer [24, 25]. Symptom burden can negatively influence cancer patients’ function, interfere with treatment outcomes [26] and affect their quality of life [27]. The higher symptom burden in Palestinian patients could be a consequence of a multitude of causes, with underlying causation less clear. Provision of cancer care in Gaza is characterised by chronic shortages of medicines and lack of access to cancer treatments, exacerbated further by difficulties experienced by patients in acquiring exit permits to access treatments outside the Gaza Strip; with cancer investigation and treatment categorised as ‘non-urgent’ applications [28]. The cancer symptom burden in Palestine is expected to increase, reaching levels that further challenge the financial and infrastructural resources of the current healthcare system, of which financial and political uncertainty exacerbate the problem [4]. This is a priority condition for developing strategies for responding to the high symptom burden in the face of limited resources.

Our findings align with previous research [27, 29], demonstrating an independent association between physical and psychological symptom burden and lower quality of life. Understanding the consequences of the burden on patients’ health and well-being is essential for minimising the severity of illness and enhancing the survival rate [30]. One approach may be the integration of routine assessment of symptom burden and quality of life to inform guidance and protocols for treatment and follow up of cancer patients. Results of a recent randomised trial of systematic monitoring of patients’ symptoms using electronic PROMs demonstrated improved clinician awareness of symptoms, better symptom management, fewer emergency visits, a better quality of life and improved overall survival in patients receiving chemotherapy for advanced cancer [31, 32].

### Implications for research and practice

Our study highlights multiple unmet needs of patients with cancer attending outpatient clinics. Alongside developing approaches to identifying the unmet needs of patients, there must be efforts to develop the capacity of services to respond. For example, this study highlighted a high prevalence of psychological symptoms, with more than half of all participants reporting feeling nervous, sad, and worrying during the week prior to participation in the study. However, psychosocial care services are not delivered as part of the Gazan healthcare system [33]. Furthermore, while routes to pain medication are being developed through the World Health Organization and treatment supplies from the Palestinian Ministry of Health in the West Bank [34], over half of the participants reported access to pain medication. However, the prevalence of pain remained high among participants. High levels of pain may be related to the incorrect beliefs and perceptions of patients with cancer and their families [35], such as avoidance of pain-relieving drugs because of their belief that drugs may lead to addiction [36]. At the health professional level, there can be common and contextually unique barriers [36, 37]. Previous research has also highlighted that healthcare professionals may not wish to prescribe pain-relieving drugs due to their lack of pain assessment skills and their false belief that drugs might cause addiction [38]. In Gaza, despite reporting good practices, research has highlighted knowledge deficits in physicians regarding cancer pain management which may hamper effective management [39]. Pain and its prevalence highlight the need for system-wide responses to improving the management of symptoms for patients with cancer, including both health professional and patient educational programmes such as public awareness of cancer signs/symptoms [40] and reducing misbeliefs about pain medications.

### Strengths and limitations

This study adopted a pragmatic approach to recruitment that enabled recruitment of a diverse sample, receiving the target required to ensure sufficient statistical power, standardising data collection and verifying self-report information against medical records. However, the study does have some limitations. As a consequence of our sampling approach, we included a heterogeneous sample, although this is reflective of those accessing and attending clinics at the two hospitals serving patients with cancer in the Gaza Strip. This included participants 13–18 years old, a population in which symptom burden and quality of life measures used in this study may not have been fully validated. To control for this, we explored the effect of age across tests, where age was not found to be significantly associated with quality of life. While standardised measures were used to determine symptom burden and quality of life, demographic and several clinical variables were self-reported by participants. Furthermore, limits in contextual data restricted additional nuance in the analysis. For example, while we sought to understand access to treatment modalities for participants, we did not determine adherence to intended regimens; on average, 30–40% of essential chemotherapy drugs are out of stock at any one time in Gaza [6]. And, while data on income was obtained it was not possible to determine whether the majority of participants receiving <2,000 NIS monthly is representative of the wider population, although efforts are underway to increase reporting around average longevity, education, and income in the State of Palestine [41].

## Conclusion

Our study highlights multiple, unresolved and problematic symptoms for participants with cancer that affect their quality of life. The widespread and high prevalence of symptoms suggests systematic assessment of symptoms as part of routine care may increase detection of problematic issues for patients. However, findings were derived from oncology services in the context of a largely fragmented and under-resourced health system, struggling to contend with a lack of effective governance, evidence-based policies, and financing. There is a need for greater advocacy and action to develop cancer care in Gaza, but any response needs to be contextually relevant and feasible in a complex environment with ongoing socio-political and economic crises.

## Supporting information

S1 Appendix Proforma used to collect data from participants. (PDF)
