# Supplementary material for: The Symptom Burden and Quality of Life in Cancer Patients in the Gaza Strip, Palestine: A Cross-Sectional Study
Source: PLoS One. 2022 Jan 13;17(1):e0262512. doi: 10.1371/journal.pone.0262512 (PMC8758072; doi:10.1371/journal.pone.0262512)
Supplement: S1 Appendix — (PDF) [file pone.0262512.s001.pdf]

Study title: **Symptom burden and quality of life of patients accessing cancer care in the Gaza Strip**

1. Hospital site: ◻ Al Rantisi Hospital ◻ EGH<br>2. The data is collected: ◻ Tasneem ◻ Mohammed ◻ Omar ◻ Saad<br>3. Participant number: _______________<br>4. Patient file ID: ____________________<br>5. Date of the interview: DD:____ MM:____YYYY:_____	6. Setting: ◻ Outpatient ◻ Other (please state) ....<br>7. Sex: ◻ Male ◻ Female<br>8. Date of birth: DD: ____ MM: ____ YYYY: ______<br>9. Governorate: ◻ North Gaza ◻ Gaza ◻ Middle ◻ Khanyounis ◻ Rafah

10. Marital status	◻ Married ◻ Single ◻ Divorced / separated / Widowed / Widower
11. Highest education qualification	◻ Less than secondary school ◻ Secondary school ◻ University degree or more
12. Residency	◻ Outside camp ◻ Inside camp
13. Monthly income (NIS)	◻ < 1000 ◻ 1000-2000 ◻ 2000-3000 ◻ > 3000 ◻ Not applicable◻ I prefer not to say

**Data item**	**Data as recorded in record**			***✓= indicate where not provided or unclear from patient (obtain from medical record after interview)***
14. Primary diagnosis leading to referral to cancer centre	◻ Brain<br>◻ Breast<br>◻ Colorectal<br>◻ Female genital and reproductive organs<br>◻ Haematological<br>◻ Head and neck	◻ Liver<br>◻Lung<br>◻ Prostate<br>◻ Other male genital and reproductive organs (excluding prostate)<br>◻ Skin<br>◻ Urological	◻ Upper gastrointestinal (upper GI)<br>◻ Soft connective tissues<br>◻ other: ....<br>◻ Unknown	
15. Date of primary cancer diagnosis	◻ Less than a month<br>◻ Within the last 3 months<br>◻ Within the last 6 months	◻ Within the last 12 months<br>◻ Within the last 2 years<br>◻ More than 2 years		
16. Date of first referral to cancer centre	MM: _____ YYYY: _________			
17. Disease stage at referral	◻ Early stages	◻ Advanced stages	◻ Unavailable / I don't know	
18. Comorbid conditions	◻ Yes ◻ No			
19. If **Yes** to comorbid conditions, please list (tick all that apply)	◻ Hypertension<br>◻ Diabetes mellitus<br>◻ Heart disease	◻ Dyslipidaemia<br>◻ Others, please identify:<br>..		
20. **Treatments** modalities received in last 3 months (tick all that apply)	◻ Chemotherapy<br>◻ Hormonal<br>◻ Radiation	◻ Surgical<br>◻ Psychological<br>◻ Other(s) (please specify):		

21. Types of pain medication accessed and the source	Medication	Hospital	Personal	
	◻ NSAID			
	◻ Paracetamol 5mg P.O			
	◻ Morphine 10mg ampule			
	◻ Morphine (P.O.) Sustained treatment (MST)			
	◻ Tramadol 100mg P.O.			
	◻ Pethidine 100mg			
	◻ Other:			
	◻ Other:			
	◻ Other:			

EORTC QLQ-C30 (Version 3)

	إطلاقاً	قليلا	بما فيه الكفاية	كثيرا جدا
1. هل لديك صعوبة ببذل مجهود جسدي شاق (متعب) مثل حمل كيس مشتريات ثقيل أو حقيبة؟	1	2	3	4
2. هل لديك صعوبة بالمشي لمسافة طويلة؟	1	2	3	4
3. هل لديك صعوبة بالمشي لمسافة قصيرة خارج البيت؟	1	2	3	4
4. هل تحتاج للبقاء بالسرير أو الكرسي خلال اليوم؟	1	2	3	4
5. هل تحتاج للمساعدة بالأكل، ارتداء الملابس، الاغتسال او استخدام المرحاض؟	1	2	3	4
خلال الأسبوع الماضي				
6. هل كنت محدود/مقيد عند القيام بعملك او نشاطات يومية أخرى؟	1	2	3	4
7. هل كنت محدود/مقيد في ممارسة هواياتك او نشاطات في أوقات الفراغ؟	1	2	3	4
8. هل كنت بحاجة للراحة؟	1	2	3	4
9. هل شعرت بالضعف؟	1	2	3	4
10. هل كنت متعب؟	1	2	3	4
11. هل شعرت بالإكتئاب؟	1	2	3	4
12. هل شعرت بالانزعاج؟	1	2	3	4
13. هل كانت لديك صعوبة بتذكر الأشياء؟	1	2	3	4
14. هل حالتك الجسدية او علاجك الطبي أثر سلبيا على حياتك العائلية؟	1	2	3	4
15. هل حالتك الجسدية او علاجك الطبي أثر سلبيا على حياتك الاجتماعية؟	1	2	3	4
16. هل حالتك الجسدية او علاجك الطبي أدى الى مشاكل مالية؟	1	2	3	4

في الأسئلة التالية الرجاء الإشارة بدائرة حول الأرقام بين 7 – 1 الأكثر ملائمة لك

17. كيف تقيم صحتك عموما خلال الأسبوع الماضي؟

1<br>سيء جدا	2	3	4	5	6	7<br>ممتاز

18. كيف تقيم /جودة حياتك عموما /مستوى حياتك عموما خلال الأسبوع الماضي؟

1<br>سيء جدا	2	3	4	5	6	7<br>ممتاز

التعليمات: وضعنا أدناه 31 عرضا. إذا حدث انك إشتكيت خلال **<u>الأسبوع الماضي</u>** من احداها، أخبرنا كم مرة أصبت به، كم كانت حدّته، والى اي حدّ <u>أزعجك أو أوجعك</u>، وهل تلقيت علاجاً لهذا العرض وكم كان هذا العلاج ناجحاً.

خلال **<u>الأسبوع الماضي</u>:**	لم أصب بهذا العرض	إذا كان الجواب نعم																	هل خضعت لعلاج مخصص للسيطرة على هذا العرض؟		إذا خضعت لعلاج، إلى أي مدى كان العلاج فعالاً في السيطرة على هذه الأعراض؟		
		كم مرة أصبت به؟			كم مرة أصبت به؟				كم كانت حدة العرض؟				كم أزعجك العرض؟									
هل أصبت بأي من الحالات التالية؟		قليلاً	بما فيه	كثيراً جدا	نادراً	أحياناً	بشكل	تقريباً باستمرار	خفيف	متوسط	حاد	حاد جداً	أبداً	قليلاً جداً	بعض الشيء	كثيراً	كثيراً جداً	نعم	لا	نجاح	نجاح بسيط	لم ينجح
1. صعوبة في التركيزز في بعض الأمور مثل قراءة الجريدة او مشاهدة التلفاز؟		1	2	3	1	2	3	4	1	2	3	4	0	1	2	3	4	1	2	1	2	3
2. الشعور بالتوتر		-	-	-	1	2	3	4	1	2	3	4	0	1	2	3	4	1	2	1	2	3
3. الشعور بالحزن		1	2	3	1	2	3	4	1	2	3	4	0	1	2	3	4	1	2	1	2	3
4. القلق		1	2	3	1	2	3	4	1	2	3	4	0	1	2	3	4	1	2	1	2	3
5. "أنا لا أشبه نفسي"		-	-	-	1	2	3	4	1	2	3	4	0	1	2	3	4	1	2	1	2	3
6. قلة النشاط		-	-	-	1	2	3	4	1	2	3	4	0	1	2	3	4	1	2	1	2	3
7. خدر /وخز في اليدين/القدمين		-	-	-	1	2	3	4	1	2	3	4	0	1	2	3	4	1	2	1	2	3
8. دوخة		-	-	-	1	2	3	4	1	2	3	4	0	1	2	3	4	1	2	1	2	3
9. ألم		1	2	3	1	2	3	4	1	2	3	4	0	1	2	3	4	1	2	1	2	3
10. هل عانيت من ألم اثر سلبا على نشاطاتك اليومية؟		1	2	3	-	-	-	-	-	-	-	-	-	-	-	-	-	-	-	-	-	-
11. صعوبة في النوم		1	2	3	1	2	3	4	1	2	3	4	0	1	2	3	4	1	2	1	2	3
12. جفاف الفم		-	-	-	1	2	3	4	1	2	3	4	0	1	2	3	4	1	2	1	2	3
13. غثيان (لعيان)		1	2	3	1	2	3	4	1	2	3	4	0	1	2	3	4	1	2	1	2	3
14. تقيؤ		1	2	3	1	2	3	4	1	2	3	4	0	1	2	3	4	1	2	1	2	3
15. تغيّر نكهة الطعام		-	-	-	1	2	3	4	1	2	3	4	0	1	2	3	4	1	2	1	2	3
16. قلة الشهية		1	2	3	1	2	3	4	1	2	3	4	0	1	2	3	4	1	2	1	2	3
17. خسارة الوزن		-	-	-	1	2	3	4	1	2	3	4	0	1	2	3	4	1	2	1	2	3
18. صعوبة في بلع الأكل		-	-	-	1	2	3	4	1	2	3	4	0	1	2	3	4	1	2	1	2	3

19. السعال/ سعلة		-	-	-	1	2	3	4	1	2	3	4	0	1	2	3	4	1	2	1	2	3
20. ضيق في التنفس		1	2	3	1	2	3	4	1	2	3	4	0	1	2	3	4	1	2	1	2	3
21. تورّم في اليدين والرجلين		-	-	-	1	2	3	4	1	2	3	4	0	1	2	3	4	1	2	1	2	3
22. التعرق		-	-	-	1	2	3	4	1	2	3	4	0	1	2	3	4	1	2	1	2	3
23. الحكة		-	-	-	1	2	3	4	1	2	3	4	0	1	2	3	4	1	2	1	2	3
24. تقرح الفم		-	-	-	1	2	3	4	1	2	3	4	0	1	2	3	4	1	2	1	2	3
25. تساقط الشعر		-	-	-	1	2	3	4	1	2	3	4	0	1	2	3	4	1	2	1	2	3
26. تغيّر في الجلد		-	-	-	1	2	3	4	1	2	3	4	0	1	2	3	4	1	2	1	2	3
27. الشعور بالانتفاخ		-	-	-	1	2	3	4	1	2	3	4	0	1	2	3	4	1	2	1	2	3
28. مشاكل في التبوّل		-	-	-	1	2	3	4	1	2	3	4	0	1	2	3	4	1	2	1	2	3
29. إسهال		1	2	3	1	2	3	4	1	2	3	4	0	1	2	3	4	1	2	1	2	3
30. إمساك		1	2	3	1	2	3	4	1	2	3	4	0	1	2	3	4	1	2	1	2	3
31. مشاكل في الرغبة أو النشاط الجنسي		-	-	-	1	2	3	4	1	2	3	4	0	1	2	3	4	1	2	1	2	3

إذا أصبت بأي حالة أخرى خلال الأسبوع الماضي، من فضلك دوّن أدناه كم أزعجتك أو آلمتك هذه الحالة.	إذا نعم، كم أزعجك العرض؟					هل خضعت لعلاج مخصص للسيطرة على هذا العرض؟		إذا خضعت لعلاج، إلى أي مدى كان العلاج فعالاً في السيطرة على هذه الأعراض؟		
	[illegible]	[illegible]	[illegible]	[illegible]	[illegible]	[illegible]	[illegible]	[illegible]	[illegible]	[illegible]
عرض:	0	1	2	3	4	1	2	1	2	3
عرض:	0	1	2	3	4	1	2	1	2	3
عرض:	0	1	2	3	4	1	2	1	2	3

أنا أقر بأني قد وافقت على أخذ هذه المعلومات مني بكامل ارادتي بما لايتعارض مع انتهاك خصوصيتي. توقيع المريض.................................
